# Investigating the mechanism of heat-shock protection in ISKNV-infected tilapia brain cell line

**DOI:** 10.1128/spectrum.02510-24

**Published:** 2025-08-12

**Authors:** Angela Naa Amerley Ayiku, Irene Amoakoh Owusu, David Verner-Jeffreys, Richard Paley, Peter Kojo Quashie, Samuel Duodu

**Affiliations:** 1 Department of Biochemistry, Cell and Molecular Biology, University of Ghana 664990 , Accra, Ghana; 2 West African Centre for Cell Biology of Infectious Pathogens, University of Ghana 635065 , Accra, Ghana; 3 College of Basic and Applied Sciences, University of Ghana 58835, Accra, Ghana; 4 WorldFish 118835, Bayan Lepas, Penang, Malaysia; 5 Cefas Weymouth Laboratory, Centre for Environment, Fisheries and Aquaculture Science 546810 , Weymouth, England, United Kingdom; Barnard College, Columbia University, New York, New York, USA

**Keywords:** heat-shock, autophagy, apoptosis, ISKNV

## Abstract

Infectious spleen and kidney necrosis virus (ISKNV) is the causative agent of a fatal disease in many fish species, resulting in mass mortalities and significant economic losses. Since its introduction to Ghana in late 2018 and in the absence of effective vaccines, the crude practice of heat-shock treatment (HST) on deliberately exposed cultured tilapia fingerlings was widely adopted by farmers to control the disease in Ghana with some apparent success. This study investigated the interplay between the expression of heat-shock proteins (HSPs) and viral replication during ISKNV infection. An *in vitro* experimental challenge study which involved deliberate infection with ISKNV and subsequent exposure of primary *Oreochromis niloticus* (tilapia) brain cell lines to HST at 48 hours post-infection was carried out. The ISKNV was confirmed by Oxford Nanopore Sequencing of the full major capsid protein (MCP), while the species identity of the cell line was confirmed by Sanger sequencing of the cytochrome C oxidase (COX1) genomic region. The test groups and control groups were screened at various time points for viral proliferation and HSP marker expression using quantitative PCR (qPCR). Exposure to heat shock significantly increased HSP 90 and 47 expressions by fourfold and sixfold, respectively, with a concomitant 10-fold decrease in viral load as compared to the non-heat-shock group. Viral apoptosis gene *ORF 005L* was significantly downregulated following increase in HSPs expression. This initial finding implies that HST may play an important role in suppressing viral replication through the apoptosis regulatory gene *ORF 005L*. This information will contribute to the understanding of the beneficial effect of heat-shock therapy used in control of the viral pathogen in aquaculture. Further studies in controlled *in vivo* experiments will give more clarity to the general effect of this treatment on tilapia growth and ISKNV persistence in infected fish populations.

**IMPORTANCE:**

Infection of tilapia by infectious spleen and kidney necrosis virus (ISKNV) is trending toward endemicity in Ghanaian lake-based tilapia farms, and it is an important fish pathogen, worldwide. This study provides a potential mechanism to explain the reported role of heat-shock in protecting fish from the negative effects of ISKNV infection. Thus, it offers strong evidence for heat-shock therapy and will lead to better disease management in Ghana and worldwide. Additionally, there are other research avenues that may lead to some therapeutic options down the line.

## INTRODUCTION

Infectious spleen and kidney necrosis virus (ISKNV) is the etiological agent implicated in several high-mortality (up to 100%) outbreaks all over the world (1–4). A key histopathological sign associated with ISKNV infection is enlarged cells in the kidney, spleen, intestine, liver, and gills (2, 5). During the first Ghana ISKNV outbreak in 2018, Megalocytivirus-like pathology included necrotic spleen, kidney, and gill tissue and pronounced megalocytes in the spleen, kidney, and lamina propria of the intestines and skeletal muscles (2). To date, ISKNV has been isolated from over 50 ornamental and food fish species across the globe, in both temperate and tropical countries (6). Species affected are primarily freshwater and brackish water fish species with economic importance such as tilapia (*Oreochromis* spp.) (7–9). The susceptibility to ISKNV disease is temperature-dependent, with infections and mortalities occurring in water temperatures above 20°C (10). Also, studies have shown that ISKNV infections are inhibited in fish at temperatures above 30°C with reduced viral load and no mortalities (11). Thus, the virus is more active within temperatures ranging from 20°C to 30°C. This implies that, for countries with tropical warmer climates where water temperatures range from 23°C to 31°C, fish susceptibility to ISKNV infection can be all year round and may even be more severe during the rainy seasons.

Although the pathogenesis of ISKNV is not well understood, the virus has been reported to enter MFF-1 host cells via the caveola-mediated endocytosis (12). Within the host cells, the internalized viral particle is capable of inducing autophagic flux (13). Recent studies have established that autophagy-related genes were significantly upregulated in ISKNV-infected Chinese perch brain (CPB) cells, aiding the viral replication (13). Autophagy plays an antiviral role by targeting viral components for lysosomal degradation (xenophagy) and initiating innate and adaptive immune system responses to viral infections (14). Several DNA viruses have been known to encode viral proteins that interact with the autophagic pathway (14, 15). These viruses make use of specific mechanisms to initiate, counteract, regulate, or inhibit autophagy in order to increase their ability to persist in their hosts (13, 14). So far, little is understood how ISKNV survives the autophagic process. Apoptosis, or programmed cell death, destroys damaged cells through a regulated autonomous process, thereby maintaining homeostasis in tissue and organs. Previous reports indicate that ISKNV induces both intrinsic and extrinsic apoptosis pathways in early- and late-stage apoptosis (16, 17). The expression of ISKNV encoded open reading frame (ORF) transcripts 111L and 005L is respectively upregulated and downregulated with increased apoptosis (17, 18). Certain heat-shock proteins (HSPs) such as HSP 90 have been demonstrated to inhibit apoptosis in tumors and transformed cells. Generally, HSPs are induced by stressors such as temperature extremes, pollutants, parasitism, anoxia, and many others (19, 20). These proteins are implicated in the general protection of stressed cells by mitigating cell damage to enhance tolerance of aquatic organisms to disease (19, 21). Notably, HSP70, HSP60, HSP90, HSC70, and GRP75 were found to be elevated in fish exposed to higher temperatures (34°C–35°C) than those living in the normal river water temperatures (25°C–29°C) (22, 23).

In this study, we investigated potential mechanisms through which heat shock apparently protects tilapia cells even during ISKNV infection. We isolated and expanded a tilapia primary brain cell line and compared the impact of heat shock on ISKNV replication and expression of HSPs and apoptotic genes.

## MATERIALS AND METHODS

### Study design

This work encompassed an *in vitro* experimental challenge on tilapia (*Oreochromis niloticus*) primary cells. For this, a tilapia brain (TiB) primary cell line was established and then exposed to a specific virus inoculum dose, with or without heat treatment. This was followed by monitoring of a range of host and viral markers by gene expression and viral load. The whole experiment was performed twice, each time with three technical replicates, to ensure reproducibility and to capture biological variations, respectively. Approval was sought from the University of Ghana Ethical Review Committee and University of Ghana Institutional Animal Care and Use Committee (UG-IACUC 007/20-21).

### Preparation of inoculum

Spleen and kidney tissue infected with ISKNV was harvested aseptically and homogenized with 10-fold volume of sterile phosphate-buffered saline (pH 7.4). The suspension was then centrifuged at 7,500 *g* for 20 minutes at 4°C, filtered through 0⋅22 µm filter membranes, and stored at −80°C until use. The 100-fold diluted virus filtrate was added to a monolayer of the primary cells and incubated for 60 minutes at 28°C. After adsorption, unattached viruses were removed, and 5 mL of Lebovitz’s L-15 medium (supplemented with 3% fetal bovine serum [FBS], 100 IU mL^−1^ penicillin, 100 µg mL^−1^ streptomycin, and 0.25 µg mL^−1^ amphotericin B) was added to flasks and incubated until extensive cytopathic effect (CPE) was observed. Virus in the culture supernatant was harvested and clarified by low-speed centrifugation, and virus stocks were stored at −80°C for 50% tissue culture infective dose (TCID_50_) determination. The full major capsid protein (*MCP*) genomic region of the virus was sequenced using Oxford Nanopore Sequencing technology (accession no. OP689650.1).

### Establishment of TiB primary cell line

Juvenile tilapia (≈50 g) were euthanized and disinfected with 75% ethanol for 2 minutes. In a biosafety cabinet, brain tissue was aseptically excised into a sterile petri dish and rinsed thrice with washing medium (Leibovitz’s L-15 + 200 IU/mL penicillin + 200 µg/mL streptomycin + 0.5 µg/mL amphotericin B) to remove blood. The tissue was minced using a sterile scalpel blade into 1 mm^3^ blocks and rinsed again in washing medium if tissue blocks were still bloody. The minced tissue was transferred into sterile falcon tubes containing dispersion medium (Leibovitz’s L-15 + 200 IU/mL penicillin + 200 µg/mL streptomycin + 0.5 µg/mL amphotericin B + 0.1% collagenase) and incubated at 28°C for 4–6 hours until tissues dispersed into single cells. The suspension was then centrifuged at 200 × *g* for 10 minutes. The supernatant was discarded, and the pellet was resuspended in growth medium (Leibovitz’s L-15 + 200 IU/mL penicillin + 200 µg/mL streptomycin + 0.5 µg/mL amphotericin B + 20% FBS + 10 ng/mL epidermal growth factor +10 ng/mL basic fibroblast growth factor) and transferred to 25 cm^2^ tissue culture flasks for incubation at 28°C. Half of the medium was changed every 4 days for 3 weeks and then subcultured to confluence. The oxidase gene of these cells was characterized using Sanger sequencing technology to confirm the species identity. The sequence has been submitted to GenBank (accession no PQ302309).

The established TiB cell line (parental cells seeded to confluence) susceptibility was compared to cultured Bluegill fry 2 (BF-2) cell line at 82nd passage by measuring the viral load at various time points. BF-2 cell lines are continuous cell lines already established for ISKNV propagation (24). Briefly, both cell lines were revived and seeded into 24 well culture plates in triplicates. Both cell lines were infected with viral inoculum with equal titer. The test groups were inoculated with a prepared inoculum of known titer and microscopically observed (100× and 200× magnification) daily till complete cell death. Daily viral load was determined using quantitative PCR (qPCR) of the viral *MCP* and the host beta-actin (β-actin) genes as target and house-keeping reference, respectively.

### TCID_50_ determination

The virus titer was determined using the TCID_50_ Reed-Muench method in a 96 well culture plate. Each well was filled with 180 µL of L-15 medium (with 10% FBS, 100 IU mL^−1^ penicillin, 100 µg mL^−1^ streptomycin, and 0.25 µg mL^−1^ amphotericin B) and 20 µL inoculum (10-fold dilution of the stock virus). Inoculation was performed in eight replicates for eight viral serial dilution steps. The plate was incubated at 28°C for 3–5 days and was observed daily for CPE to determine the TCID_50_. The multiplicity of infection (MOI) was determined from the viral titer.

### *In vitro* challenge experimental design: ISKNV and TiB host interaction

**Fig 1:**
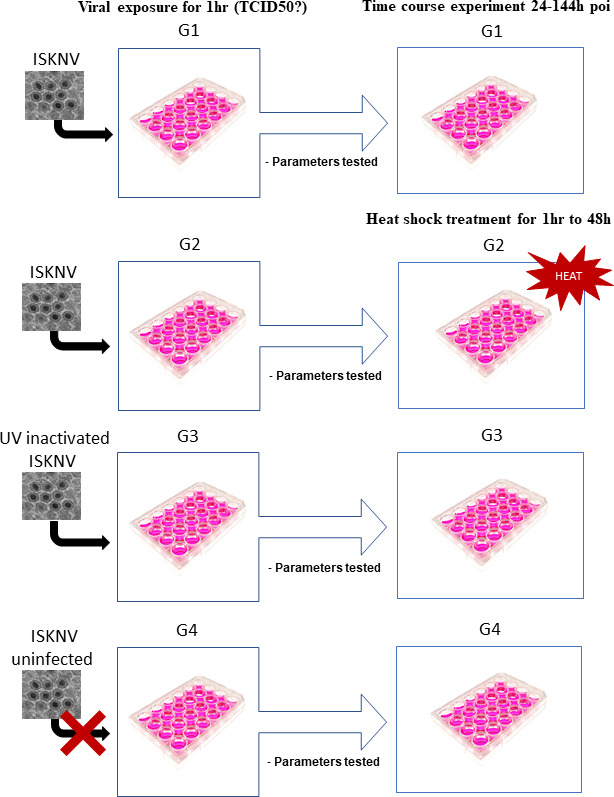
A schematic of the hyperthermia challenge experiment using the established primary TiB cell lines.

Two test groups of TiB cells were infected with ISKNV in triplicate group (MOI = 0.02) (Fig. 1). Test group G1 was not heat-shocked at any time point till the end of the experiment, and test group G2 was heat-shocked at 48 hours post-inoculation (poi). Two control groups included in the challenge experiment were also triplicated. The control group G3 was mock-infected (UV-inactivated virus), and G4 was uninfected. After 2 hours of adsorption at 28°C, the inoculum was removed, and the cells were carefully washed with Hanks’ Balanced Salt Solution. The cells were then covered with 100 µL of maintenance medium (Table S1). Before 48 hours, one ISKNV-infected test group was exposed to gradually increasing heat in a separate incubator, ramped up from 28°C up to 38°C, at a rate of 2°C/10–12 min, for 1 hour. Samples were then collected from the heat-treated and untreated groups, after which the culture was returned to the incubator at 28°C. One set of uninfected cells also underwent this heat exposure. The test and control groups were sampled at time points 24, 48, 72, 96, 120 hours poi. At each time point, three parallel samples were pooled as a biological replicate. Images of the cells were captured for each group at every time point using the AmScope digital camera to check for ISKNV CPE during the hyperthermia experimental challenge.

### Quantification of ISKNV copies in cells and determination of extracellular viral yield

The culture media in each well was pipetted without disrupting the monolayer. This was then centrifuged at 1,000 rpm for 5 minutes, and 50 µL of the supernatant was transferred into fresh Eppendorf tubes. Each test group was sampled at each time point, and DNA was extracted using the QIAamp DNA Mini Kit (Qiagen). Total DNA was extracted from the culture media or cells according to the manufacturer’s protocol. The samples were incubated with 10 µL of proteinase K and 100 µL of lysis buffer at 56°C for 10 minutes. After which total nucleic acids were extracted and stored at −20°C until further use. A SYBR Green-based qPCR of the *MCP* target (Table 1) was used to determine the viral copies. A 20 µL reaction mix consisting of 10 µL of 2 × iTaq Universal SYBR Green Supermix (Bio-Rad), 0.6 µL of both forward and reverse primers, 6.8 µL of molecular grade water, and 1 µL of DNA template was prepared. The temperatures and qPCR cycle conditions are as follows: 95°C (3 minutes), one cycle; 95°C (10 seconds)/60°C (1 minute)/95°C (30 seconds), 45 cycles. To calculate for viral copy numbers, the concentrations of the viral titer standards were determined initially by nanodrop measurement, and then a 1/10 dilution of 20 ng to 2 ng concentration was done, followed by an eight-step 1/10 serial dilution from 2 ng to 200 ag. The following formula was used to determine the viral copies in each dilution: number of copies = (ng × [6.022 × 10^23^])/(length × [1 × 10^9^] × 650) where ng is the amount of DNA in nanograms, 6.022 × 10^23^ is Avogadro’s number, and length is the length of the DNA fragment in base pairs and multiplied by 1 × 10^9^ to convert to nanograms. To generate a standard curve, the eight dilutions were used as templates to run the SYBR Green qPCR. A graph of Ct mean values against log-transformed viral copy numbers for each dilution was used to determine the slope and intercept. The viral copy numbers of each test group were then extrapolated from the Ct values using the formula => 10^((observed Ct Mean-Y Intercept)/(Slope)) where intercept = 42.905 and slope = −3.743 (Table S2; Fig. S1).

**TABLE 1:** Primers used for HSPs and ORF 005L expression and ISKNV DNA detection

Gene	Primers	Sequence	Reference
*MCP*	Forward primer	AGTCAAGGAACTCGCTGGTG	(25)
	Reverse primer	GTGACCTACTTTGCCCGTGA	
*HSP 60*	Forward primer	CGGAAGATGTGGATGGAGA	(26)
	Reverse primer	GACGGCCACTCCATCTG	
*HSP 47*	Forward primer	CACTGGGATGAGAAGTTCCA	(26)
	Reverse primer	AAGGAAAATGAAGGGATGGTC	
*HSP 90*	Forward primer	ACCAAACACAACGATGACGA	(26)
	Reverse primer	CCGATGAACTGGGAGTGTTT	
*ORF 005L*	forward primer	GTAGATGATAATGACGCTGTGAAGG	(17)
	Reverse primer	GTGTCGTGCTCGGGTGTTG	
*β-actin*	Forward primer	TGGGGCAGTATGGCTTGTATG	(26)
	Reverse primer	CTCTGGCACCCTAATCACCTCT	

### Expression of HSPs and apoptotic genes

Total RNA was extracted from TiB cells using the QIAamp RNA Mini Kit (Qiagen). The expression of β-actin, HSPs 47, 60, and 90 and ORF 005L mRNA was quantified by reverse transcription (RT)-qPCR at each time point. The RT-qPCR was carried out on all samples using the sets of primers in Table 1. The reaction mix was formulated as follows: 6.8 µL nuclease-free water, 10 µL of 2× Luna universal one step, 1 µL of 20× Luna Warmstart RT enzyme, 0.8 µL of both 10 µM forward and reverse primers, and 1 µL of the RNA template. The cycling conditions involved RT at 55°C for 20 minutes, an initial denaturation step at 95°C for 3 minutes, then 40 cycles of denaturation at 95°C for 10 seconds, and annealing and extension at 50°C for 30 seconds. All analysis was done on three merged biological replicates. HSP expression analysis of the PCR data were computed using the “comparative *C*_*T*_ Method (ΔΔ*C*_*T*_ Method)” and the fold difference of HSP markers and viral apoptosis genes at each time point relative to expression levels at 24 hours poi was calculated using the 2^−∆∆*CT*^ formula. The mean Ct values and standard deviations for each target gene and the β-actin endogenous control from three replicates were used for the calculation. To normalize the data, Δ*C*_*T*_ was calculated by subtracting the mean Ct value of the β-actin gene from that of the target genes for both heat-shock treated and untreated groups (Δ*C*_*T*_ = *C*_*T* target_ – *C*_*T* reference_). The variance of the Δ*C*_*T*_ was calculated from the standard deviations of the target and endogenous gene (reference gene) values using the formula: *s* =√ (*s*1^2^ + *s*2^2^) where *s* = standard deviation. To determine the gene transcript expression fold difference between two time points within the treated and untreated groups, the ΔΔ*C*_*T*_ was first calculated by using the formula: ΔΔ*C*_*T*_ = Δ*C*_*T*_ test sample at time point A – Δ*C*_*T*_ test sample at time point B. To get the true fold difference, the log_2_ of the ΔΔ*C*_*T*_ value was calculated. This was to even out the scales of transcript expression (2^−∆∆*CT*^). The upper and lower limits were determined using the formula: 2^−∆∆*CT*+*S*^ and 2^−∆∆*CT*−*S*^, respectively (Tables S3 and S4). Gene transcript expression of fold change values above 2 was considered upregulated and below 2, downregulated (27–29).

### Statistical analysis

Multiple *t*-test analyses at 95% confidence interval (CI) (GraphPad Prism 8.0.2) were used to test the significant difference in fold change at each time point between the treated and untreated groups. Additionally, the statistical significance between the expression levels of HSP 90/HSP 47 and ORF 005L transcripts was determined using multiple *t*-test. The *P-*values are represented in Tables S5 and S6.

## RESULTS

### Susceptibility of TiB primary cell line as compared to BF-2 continuous cell line

**Fig 2:**
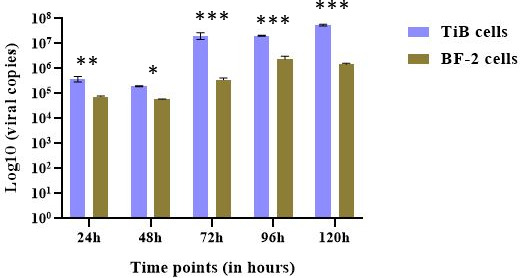
ISKNV extracellular viral titers at various time points (X axis) of TiB and BF-2 cell lines post-infection. The viral titers extrapolated from qPCR Ct values were log transformed (*Y* axis). Inoculum titer ⁓1.12 × 10^6^ TCID_50_/mL with an MOI of 0.02. *P < 0.05, **P < 0.005, and ***P < 0.0005.

Following TCID determination (with an approximate titer value of 1.12 × 10^6^ TCID_50_/mL), all inoculation was done using an MOI of 0.02. At 24 and 48 hours poi, the cell morphology did not change appreciably, with no CPE observed. Typical CPE as described in Dong et al. (2008) (30) was visible from 48 hours poi. Attached cells became rounded and eventually detached from the plate. At 72 hours poi, CPE (rounded and detached cells) became more evident in the infected wells, compared to uninfected wells; this became even more pronounced by 96 hours poi. Interestingly, virus-infected TiB primary cells typically showed more pronounced CPE than BF-2 cells. The TiB monolayer was depleted by 120 hours poi, while about 50% of BF-2 cells remained attached (Fig. S2). The CPE progression correlated with viral titers recorded each day at the various time points. Viral titer dipped by approximately two- and 1.5-fold at 48 hours poi for TiB and BF-2 infected cells, respectively. At 72 hours poi, the viral titers increased by a 100-fold in TiB cells but increased only fivefold in BF-2 cells. The experiment was terminated at 120 hours poi. On average, viral titer was approximately 150-fold higher in TiB cell and 30-fold higher in BF-2 cell at 120 hours poi (Fig. 2).

### Hyperthermia challenge experiment using established TiB primary cell line infected with ISKNV

**Fig 3:**
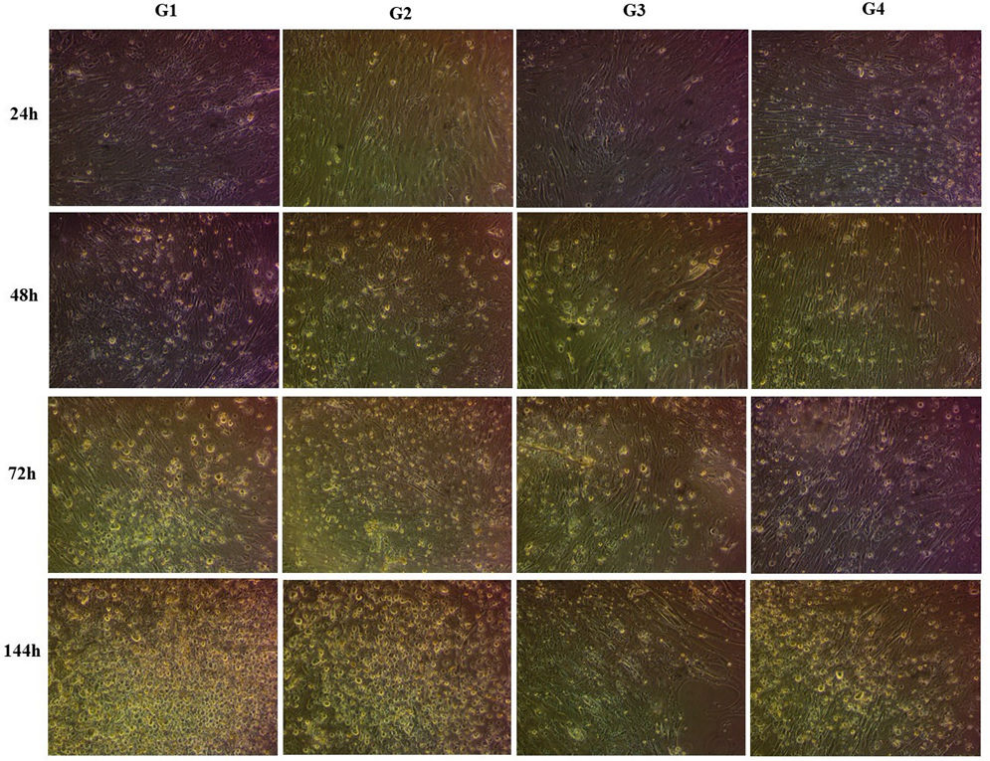
Cell culture images showing CPE (cell rounding and megalocytosis) over the time course (24–120 hours poi) of the *in vitro* experimental challenge. (G1) test group that was infected but not heat-shocked, (G2) test group that was infected and heat-shocked at 48 h poi, (G3) control group mock-infected and (G4) control group that was not infected nor heat-shocked. Magnification: 3,584 × 2,748

ISKNV-infected TiB cells were used for this experiment. At 24 hours poi, which is the adsorption and viral entry stage (13, 16), CPE was unremarkable for all four groups. At 48 hours when the virus is expected to have been internalized, initiating replication and transcription (13, 16), CPE was still unremarkable for all groups. However, CPE became obvious after 72 hours for all ISKNV-infected groups and by 144 hours, the CPE of non-heat-shocked test group G1 was relatively more pronounced than the heat-shocked and control groups (G2, G3, and G4) (Fig. 3).

**Fig 4:**
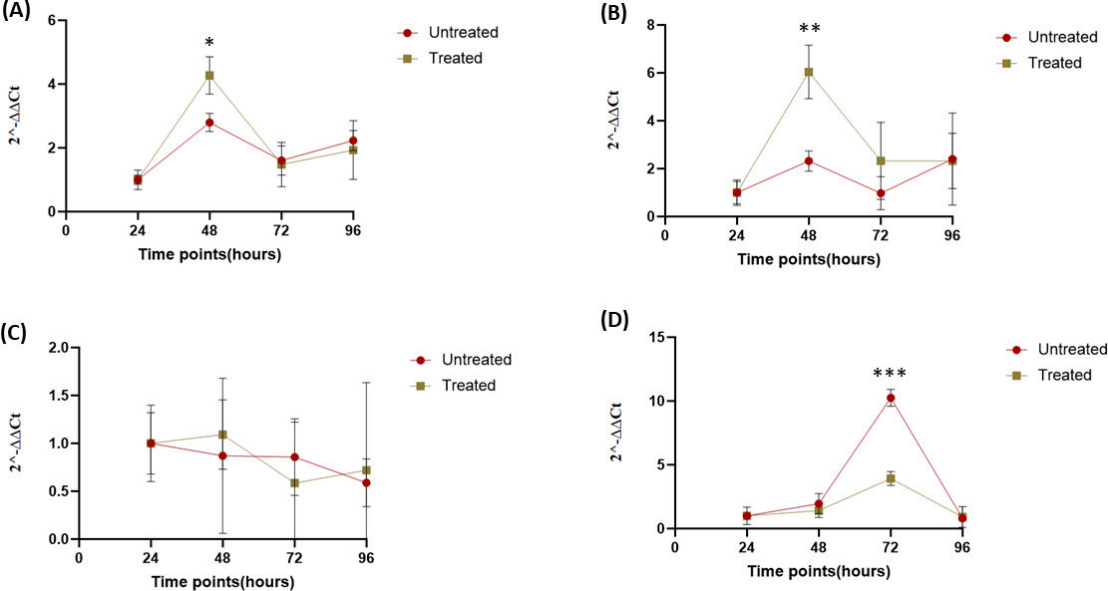
Differential expression at various time points poi between the heat-treated (Treated) and non-heat-treated (Untreated) test groups (**A**) HSP 90, (**B**) HSP 47, (**C**) HSP 60, and (**D**) ORF 005L. Heat shock was performed for 1 hour up to 48 hours poi, and samples were collected right after the procedure. The fold change from one timepoint to the next for both groups was used to generate the graphs above. A gene transcript expression fold change >2 was considered upregulated and <2, downregulated. HSP 60 was not upregulated at any time point throughout the experiment. Gene transcript HSP 90, HSP 47, and ORF 005L were upregulated and downregulated at specific time points. *P < 0.05, **P < 0.005, and ***P < 0.0005.

**Fig 5:**
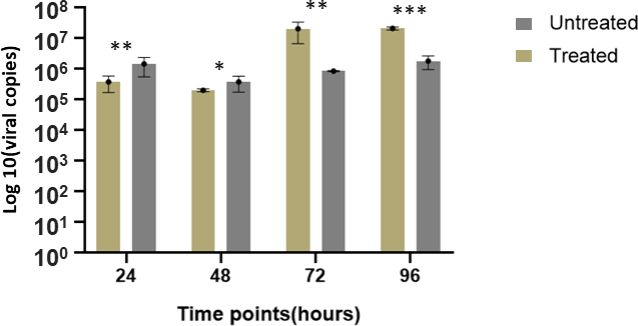
ISKNV extracellular viral titers at various time points (X axis) post-infection between the heat-treated (Treated) and non-heat-treated test groups (Untreated). The viral titers extrapolated from qPCR Ct values were log transformed (Y axis). Inoculum titer ⁓ 1.12 × 10^6^ TCID_50_/mL with an MOI of 0.02. *P < 0.05, **P < 0.005, and ***P < 0.0005.

**Fig 6:**
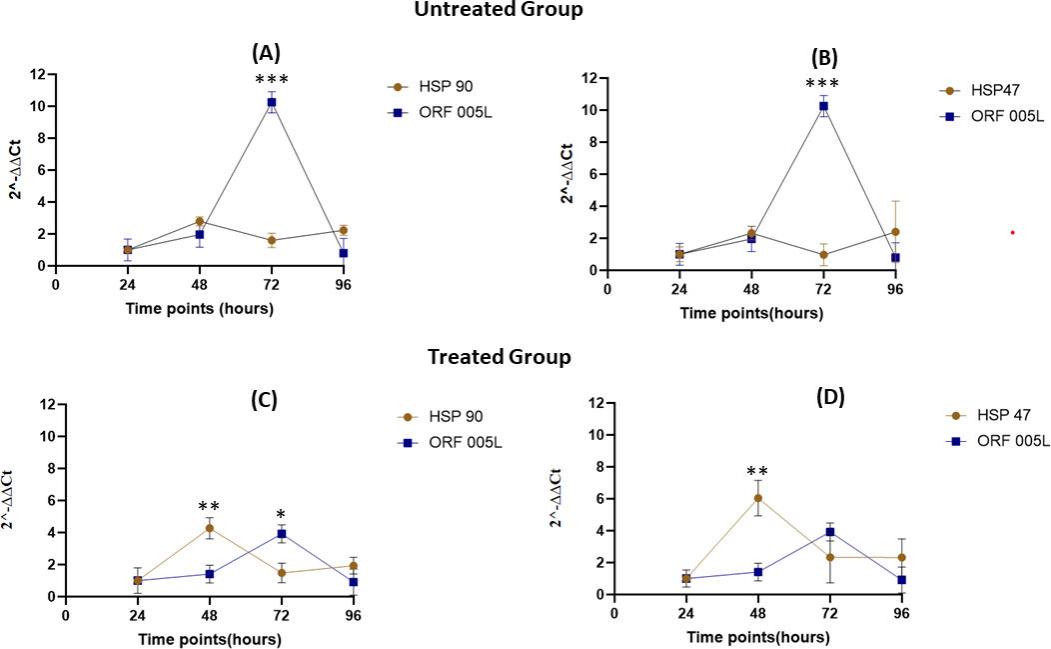
Comparing gene transcript expression between HSP 90 and ORF 005L (**A and C**) and between HSP 47 and ORF 005L (**B and D**) for both the “untreated” and “treated” test groups to show the inverse relationship at specific time points. For the “treated” group, HSP 90 was upregulated, while ORF 005L was downregulated at 48 hours poi (*P* = 0.004624). This is reversed by time point 72 hours poi (*P* = 0.007041). The same pattern is seen with HSP 47 at 48 (*P* 0.002998) and 72 hours poi (*P* = 0.179486), even though the difference at 72 h poi is not as significant. In the “untreated” group, the expression levels of HSP 90 and HSP 47 transcripts remained largely unchanged throughout the experiment, showing no significant upregulation. ORF 005L was significantly upregulated at 72 poi compared to its expression at the same time point in the treated group (A: *P* = 0.000049 and B: *P* = 0.000074). The fold change values were used for this analysis (*Y* axis). *= *P* < 0.05, **= P < 0.005, ***P < 0.0005.

For heat-shock gene expression analysis, the untreated group (cells infected but not heat-shocked) was compared with the treated group that was heat-shocked for an hour at 48 hours poi. HSP overexpression was almost instant after heat-shocking. The results showed a six- and fourfold increase in expression levels of HSP 47 and 90, respectively, after heat treatment (Table S3). Both HSP 90 (*P* = 0.017632) and HSP 47 (*P* = 0.005710) were significantly upregulated, whereas HSP 60 showed a very minimal increase (Table S5; Fig. 4). The viral load over the time course for each group also varied (Fig. 5). The viral load plummeted approximately twofold for untreated and fourfold for treated TiB cells at 48 hours poi. There was an appreciable recovery of viral titers with the untreated (100-fold increase) compared to the treated group (2.5-fold increase) at 72 hours poi (Fig. 5). Viral load of untreated group remained over 100-fold higher than the treated group at 96 hours poi. Viral encoded ORF 005L gene was significantly upregulated (*P* = 0.00027) at 72 hours poi for untreated, and relatively downregulated for the treated group with ≈5-fold difference between the untreated and treated (Fig. 4). There seemed to be a link between ORF 005L expression and viral titers at each time point for both untreated and treated groups. On the other hand, there seemed to be an inverse relationship between the expression of HSPs 90 and 47 with ORF 005L at 48 and 72 hours poi after heat-shock treatment (HST) (Fig. 6).

## DISCUSSION

Highly infectious diseases such as those caused by ISKNV are one of the major challenges faced by the aquaculture industry in Ghana and worldwide. The Nile tilapia is a highly susceptible ISKNV host species. Up to 95% mortality was recently reported on tilapia culture farms in Ghana (2) with the virus still persisting (25). Viral diseases lacking effective chemo- or immunotherapy are usually managed with preventive measures such as strict enforcement and adherence to biosecurity measures and responsive measures including stamping out and movement restrictions. Unfortunately, when the virus becomes endemic and broadly distributed, as is now the case in Lake Volta, Ghana (25), these control measures become less effective. A recent study showed that apart from vaccination, one alternative method gaining traction among tilapia farmers is HST, applied variably across farms (25). Although this method is not well optimized, it has on occasion appeared effective in reducing ISKNV-related mortality in fry population and increasing relative productivity (25).

Searching for effective therapeutic agents against this viral pathogen will require understanding of its pathogenesis. However, development of cell lines for pathogenesis research for this virus was initially challenging, as commercially available cell lines were not permissive to all ISKNV genotypes (4, 31, 32). Several cell lines such as the grunt fin have been established; however, the viral titers tend to decrease or deplete after serial passages (33, 34). Chinese Perch (*Siniperca chuatsi*) brain (CPB) and bluegill fry (*Lepomis macrochirus*) (BF-2) are continuous cell lines which demonstrate relatively higher and stable virus propagation rates and have been used extensively to study ISKNV pathogenesis, viral life cycle, and vaccine development (13, 16, 24, 35–38). As host symptoms and pathologies differ with species type, it is conceivable that pathogenesis also differs. The most ideal situation will be to use natural or specific host-derived cell lines for viral pathogenesis studies. Presently, there is no commercially available continuous *O. niloticus* cell line available to study ISKNV virus-host interactions (12, 13, 34, 36, 39). Previously, TiB and liver cell lines had been developed for successful propagation of tilapia lake virus (40). This study demonstrated that the TiB cell line is also susceptible to *in vitro* infection by ISKNV infection and may thus be a good candidate for tilapia-ISKNV interaction research.

HSP expression has been reported to be elevated whenever aquatic organisms are exposed to extreme water temperatures and is often used as a marker of stressful environmental conditions in certain aquatic systems (20). The practice of HST generally involves ISKNV pre-exposure, followed by exposure to temperatures appreciably higher than the ambient temperature of 25°C–28°C (25). In a recent study, prolonged holding with non-lethal hyperthermia was used to enhance survival of tilapia in intraperitoneal ISKNV inoculated fry (11). Our study investigated the potential mechanisms of enhanced survival *in vitro* and demonstrated that HSPs were already upregulated after infection *in vitro* but became significantly overexpressed after exposure to a gradual temperature increment up to 10°C higher than the ambient temperature of 28°C for an hour. This significant fold change in HSPs, specifically for HSP 90 and 47, was associated with decreasing extracellular viral titers in the heat-shocked experimental group G2 (the test group that was infected and heat-shocked at 48 hours poi) (Fig. 4 and 5). Under environmental and cellular stresses, HSPs functionally protect other proteins by regulating protein folding and support the structure of properly folded proteins (41). Some HSPs have an antiviral effect and can do so by inhibiting viral proliferation via the interaction and activation of immune pathways for host protection (41). Small HSPs such as the ones screened for in this study are produced in large quantities in response to stress and assemble complexes to modulate apoptosis (41, 42). Our data suggest that the functional role of *HSP 90*, *60,* and *47* may have been enhanced and that this enhancement may have contributed to the reduction in viral replication. The induction of autophagic flux and early apoptosis, as a survival mechanism to escape degradation at end stage autophagy, has been demonstrated for some viruses (13, 16).

It is speculated that inhibition of apoptosis may interfere with viral release from infected cells after replication. The virus-encoded *ORF 111L* was found to be similar to the tumor necrosis factor receptor-associated factor, which is essential for apoptotic signal transduction in fish, mice, and mammals and also directly interacts with zebrafish TNF receptor type 1 associated death domain protein. During an *in vivo* challenge experiment, *ORF 111L* overexpression microinjected into zebrafish embryos resulted in increased apoptosis and was associated with significant caspase-8 upregulation and activation (18). Likewise, *ORF 005L* protein contains a region similar to the catalytic domain of CTD-like phosphatases. A knockdown of this gene significantly reduced apoptosis of Mandarin fish fry-1 cells induced by ISKNV infection (17). The downregulation of the viral apoptotic gene *ORF 005L* expression at time point 72 hours, 24 hours after HSP 90, 60, and 47 upregulation, suggests that HSP may be associated with the inhibition of the viral apoptosis gene expression. These findings emphasize the potential functional role of *HSP 90* and *47* to interfere with the viral life cycle. Although this study did not directly demonstrate the expression of the HSP proteins, the overexpression of mRNA after heat shock indicates that the exposure to higher temperatures does in fact promote transcription of HSPs.

The farmers usually expose the tilapia host to the virus prior to HST. Although ISKNV infection somewhat promoted HSP mRNA expression at 48 hours poi, this did not slow the progression of the infection nor reduce the viral load. Viruses have been shown to hijack HSPs in their host cells; therefore, it is possible that the overexpression of HSPs via heat shock may have overcome the hijacking activity of the viral pathogen.

Based on this study, HST may slow the progression of the disease significantly without clearing the virus, meaning that this practice may lead to persistence of the virus or the HST may buy enough time for the classical immune system to take over, reducing viral load and disease. More studies will be required to understand the long-term effect of the HST on infected tilapia.

### Conclusion

This study demonstrated the effect of short-term HST on ISKNV replication in a primary tilapia cell line. The elevation of HSP due to hyperthermia may have played a protective role in ensuring the integrity of infected tissue, slowing disease progression and severity. The data from this study serves as a baseline for further understanding of mechanisms involved in the use of HST as a control measure. Since the virus is currently endemic in some countries already, HST may be the best hope for many afflicted farmers. However, further studies into protein expression and the binding activity of HSPs during viral infection should give more in-depth information. A series of *in vivo* challenge experiments is recommended for further investigation into the mechanism and efficacy of this management intervention and options for best practice.

## SUPPLEMENTAL MATERIAL

Tables S1 to S6 spectrum.02510-24-s0001.docx Media for primary cell culture, estimation of viral copy numbers, and gene expression.

Figures S1 and S2 spectrum.02510-24-s0002.docx Extrapolation of viral copy numbers and ISKNV infected TiB and BF2 cells at different time points.

ASM does not own the copyrights to Supplemental Material that may be linked to, or accessed through, an article. The authors have granted ASM a non-exclusive, world-wide license to publish the Supplemental Material files. Please contact the corresponding author directly for reuse.
