# Supplementary material for: Investigating the mechanism of heat-shock protection in ISKNV-infected tilapia brain cell line
Source: Microbiol Spectr. 2025 Aug 12;13(9):e02510-24. doi: 10.1128/spectrum.02510-24 (PMC12403615; doi:10.1128/spectrum.02510-24)
Supplement: Tables S1 to S6 — Media for primary cell culture, estimation of viral copy numbers, and gene expression. [file spectrum.02510-24-s0001.docx]

Table S1. Primary cell culture media composition for the various processing stages.

| **Name of Medium** | **Composition** |
| --- | --- |
| Washing medium | Leibovitz's L-15 + 200 IU/mL penicillin + 200 µg/mL streptomycin + 0.5 ug/mL amphotericin B |
| Dispersion medium | Leibovitz's L-15 + 200 IU/mL penicillin + 200 µg/mL streptomycin + 0.5 ug/mL amphotericin B + 0.8mL Tris solution + 2.5mL sodium bicarbonate (7.5%) + 0.1% collagenase |
| Growth medium | Leibovitz's L-15 + 200 IU/mL penicillin + 200 µg/mL streptomycin + 0.5 ug/mL amphotericin B + 0.8 mL Tris solution + 2.5 mL sodium bicarbonate (7.5%) + 20% FBS + 10 ng/mL EGF + 10 ng/mL BFGF |
| Maintenance medium-1 | Leibovitz's L-15 + 200 IU/mL penicillin + 200 µg/mL streptomycin + 0.5 µg/mL amphotericin B + 0.8mL Tris solution + 2.5mL sodium bicarbonate (7.5%) + 20% FBS |
| Maintenance medium-2 | Leibovitz's L-15 + 200 IU/mL penicillin + 200 µg/mL streptomycin + 0.5 µg/mL amphotericin B + 0.8mL Tris solution + 2.5mL sodium bicarbonate (7.5%) + 10% FBS |

### Table S2. Extrapolating copy numbers using the formula =>10^((observed Ct Mean-Y Intercept)/(Slope)) where intercept= 42.905 and Slope= -3.743

| Sample Name | Target Name | Ct Mean | Ct SD | Copy No.-SD | Copy No.+SD | Copy No. |
| --- | --- | --- | --- | --- | --- | --- |
| G1T1 | MCP | 22.052 | 0.407 | 290,023 | 478,707 | 372,607 |
| G1T2 | MCP | 23.075 | 0.069 | 190,326 | 207,135 | 198,553 |
| G1T3 | MCP | 15.577 | 0.511 | 14,611,797 | 27,391,815 | 20,006,090 |
| G1T4 | MCP | 15.526 | 0.076 | 19,697,652 | 21,632,395 | 20,642,369 |
| G1T5 | MCP | 13.950 | 0.116 | 50,696,502 | 58,455,354 | 54,437,872 |
| G4T1 | MCP | 19.854 | 0.468 | 1,079,980 | 1,920,728 | 1,440,260 |
| G4T2 | MCP | 22.063 | 0.394 | 290,324 | 471,467 | 369,970 |
| G4T3 | MCP | 20.735 | 0.021 | 826,545 | 848,519 | 837,460 |
| G4T4 | MCP | 19.520 | 0.341 | 1,433,909 | 2,181,780 | 1,768,749 |
| BFT1 | MCP | 24.642 | 0.081 | 72,054 | 79,604 | 75,735 |
| BFT2 | MCP | 25.077 | 0.081 | 55,118 | 60,924 | 57,948 |
| BFT3 | MCP | 22.134 | 0.281 | 297,975 | 421,219 | 354,278 |
| BFT4 | MCP | 19.048 | 0.471 | 1,770,223 | 3,159,421 | 2,364,927 |
| BFT5 | MCP | 19.820 | 0.158 | 1,333,867 | 1,620,649 | 1,470,282 |

### Table S3. HSP expression analysis using the Comparative CT Method (ΔΔCT Method)

| **Sample ID** | **Target** | **Ct mean** | **Ct SD** | **Ct SD^2^** | **Ct mean (BA)** | **Ct SD(BA)** | **BA Ct SD^2^** | **sum Ct SD^2^** | **∆Ct** | **∆CtSD** | **∆∆Ct** | **∆∆Ct+∆CtSD** | **∆∆Ct-∆CtSD** | **2^-(∆∆Ct+∆CtSD)^** | **2^-∆∆Ct-∆CtSD^** | **2^-∆∆Ct^** |
| --- | --- | --- | --- | --- | --- | --- | --- | --- | --- | --- | --- | --- | --- | --- | --- | --- |
| G1T1 | H90 | 22.559 | 0.027 | 0.001 | 29.960 | 0.139 | 0.019 | 0.020 | -7.401 | 0.141 | 0 | 0.141 | -0.141 | 0.907 | 1.103 | 1 |
| G4T1 | H90 | 22.322 | 0.016 | 0.000 | 31.010 | 0.306 | 0.094 | 0.094 | -8.688 | 0.306 | 0.000 | 0.306 | -0.306 | 0.809 | 1.237 | 1 |
| G1T2 | H90 | 22.472 | 0.030 | 0.001 | 31.358 | 0.284 | 0.081 | 0.082 | -8.886 | 0.286 | -1.485 | -1.199 | -1.771 | 2.296 | 3.412 | 2.799106 |
| G4T2 | H90 | 22.209 | 0.464 | 0.215 | 32.992 | 0.362 | 0.131 | 0.346 | -10.783 | 0.589 | -2.094 | -1.506 | -2.683 | 2.840 | 6.422 | 4.270565 |
| G1T3 | H90 | 22.460 | 0.260 | 0.067 | 30.546 | 0.376 | 0.141 | 0.209 | -8.086 | 0.457 | -0.685 | -0.228 | -1.142 | 1.171 | 2.208 | 1.608133 |
| G4T3 | H90 | 22.391 | 0.281 | 0.079 | 31.646 | 0.637 | 0.406 | 0.485 | -9.255 | 0.697 | -0.567 | 0.130 | -1.264 | 0.914 | 2.401 | 1.481207 |
| G1T4 | H90 | 22.427 | 0.254 | 0.065 | 30.988 | 0.189 | 0.036 | 0.100 | -8.561 | 0.317 | -1.160 | -0.843 | -1.477 | 1.794 | 2.783 | 2.234716 |
| G4T4 | H90 | 22.625 | 0.185 | 0.034 | 32.263 | 0.904 | 0.816 | 0.851 | -9.638 | 0.922 | -0.950 | -0.028 | -1.872 | 1.019 | 3.661 | 1.931902 |
| G1T1 | H47 | 30.129 | 0.443 | 0.196 | 29.960 | 0.139 | 0.019 | 0.216 | 0.169 | 0.464 | 0 | 0.464 | -0.464 | 0.725 | 1.380 | 1 |
| G4T1 | H47 | 30.834 | 0.443 | 0.196 | 31.010 | 0.306 | 0.094 | 0.290 | -0.176 | 0.538 | 0 | 0.538 | -0.538 | 0.688 | 1.452 | 1 |
| G1T2 | H47 | 30.309 | 0.313 | 0.098 | 31.358 | 0.284 | 0.081 | 0.178 | -1.049 | 0.422 | -1.218 | -0.795 | -1.640 | 1.736 | 3.117 | 2.325971 |
| G4T2 | H47 | 30.221 | 1.055 | 1.113 | 32.992 | 0.362 | 0.131 | 1.244 | -2.772 | 1.116 | -2.595 | -1.480 | -3.711 | 2.789 | 13.092 | 6.042455 |
| G1T3 | H47 | 30.745 | 0.576 | 0.332 | 30.546 | 0.376 | 0.141 | 0.473 | 0.200 | 0.688 | 0.031 | 0.719 | -0.657 | 0.608 | 1.577 | 0.978933 |

### Table S4. ORF 005 expression analysis using the Comparative CT Method (ΔΔCT Method)

| **Sample** | **Ct Mean ORF005L** | **Ct SD ORF005L** | **Ct SD^2^** | **Ct Mean BA** | **Ct SD BA** | **CtSD^2^** | **∑CtSD^2^** | **∆Ct** | **∆Ct SD** | **∆∆Ct** | **∆∆Ct + ∆Ct SD** | **∆∆Ct - ∆Ct SD** | **2^-∆∆Ct^** | **2^-(∆∆Ct + ∆Ct SD)^** | **2^-(∆∆Ct - ∆Ct SD)^** |
| --- | --- | --- | --- | --- | --- | --- | --- | --- | --- | --- | --- | --- | --- | --- | --- |
| G1T1 | 20.716 | 0.539 | 0.290 | 29.999 | 0.020 | 0.000 | 0.290 | -9.283 | 0.539 | 0.000 | 0.539 | -0.539 | 1.000 | 0.688 | 1.453 |
| G1T2 | 20.489 | 0.323 | 0.105 | 30.745 | 0.103 | 0.011 | 0.115 | -10.255 | 0.339 | -0.972 | -0.633 | -1.312 | 1.962 | 1.551 | 2.483 |
| G1T3 | 16.705 | 0.046 | 0.002 | 30.319 | 0.588 | 0.346 | 0.348 | -13.614 | 0.590 | -4.331 | -3.741 | -4.921 | 20.122 | 13.370 | 30.285 |
| G1T4 | 16.834 | 0.004 | 0.000 | 30.139 | 0.123 | 0.015 | 0.015 | -13.305 | 0.123 | -4.022 | -3.899 | -4.145 | 16.245 | 14.918 | 17.916 |
| G4T1 | 20.716 | 0.539 | 0.290 | 30.929 | 0.021 | 0.000 | 0.291 | -10.213 | 0.539 | 0.000 | 0.539 | -0.539 | 1.000 | 0.688 | 1.453 |
| G4T2 | 21.269 | 0.297 | 0.088 | 31.440 | 0.799 | 0.638 | 0.726 | -10.171 | 0.852 | 0.041 | 0.894 | -0.811 | 0.972 | 0.538 | 1.753 |
| G4T3 | 19.259 | 0.008 | 0.000 | 31.404 | 0.838 | 0.702 | 0.702 | -12.145 | 0.838 | -1.932 | -1.094 | -2.770 | 3.816 | 2.135 | 6.822 |
| G4T4 | 19.914 | 0.077 | 0.006 | 31.935 | 0.289 | 0.083 | 0.089 | -12.021 | 0.299 | -1.808 | -1.509 | -2.107 | 3.502 | 2.847 | 4.308 |

**Table S5. Test of significance between “Untreated” and “Treated” groups using the multiple t test statistical method for grouped data.** **(N=6)**

| **Gene** | **Time**  **point** | **Significant?** | ***P-value*** | **x̄ fold change**  **Untreated** | **x̄ fold change Treated** | **Difference** | **SE of Difference** | **t ratio** | **df** |
| --- | --- | --- | --- | --- | --- | --- | --- | --- | --- |
| ***HSP 90*** | 24 | No | >0.999999 | 1.000 | 1.000 | 0.000 | 0.1945 | 0.000 | 4.000 |
|  | 48 | **Yes** | 0.017632 | 2.799 | 4.271 | -1.472 | 0.3780 | 3.894 | 4.000 |
|  | 72 | No | 0.804878 | 1.608 | 1.481 | 0.1270 | 0.4812 | 0.2639 | 4.000 |
|  | 96 | No | 0.618938 | 2.235 | 1.932 | 0.3030 | 0.5629 | 0.5383 | 4.000 |
| ***HSP 47*** | 24 | No | >0.999999 | 1.000 | 1.000 | 0.000 | 0.4102 | 0.000 | 4.000 |
|  | 48 | **Yes** | 0.005710 | 2.326 | 6.042 | -3.716 | 0.6888 | 5.395 | 4.000 |
|  | 72 | No | 0.251394 | 0.9789 | 2.331 | -1.352 | 1.009 | 1.340 | 4.000 |
|  | 96 | No | 0.954782 | 2.405 | 2.326 | 0.07823 | 1.297 | 0.06034 | 4.000 |
| ***HSP 60*** | 24 | No | >0.999999 | 1.000 | 1.000 | 0.000 | 0.2961 | 0.000 | 4.000 |
|  | 48 | No | 0.686029 | 0.8700 | 1.093 | -0.2231 | 0.5130 | 0.4350 | 4.000 |
|  | 72 | No | 0.565678 | 0.8572 | 0.5857 | 0.2715 | 0.4343 | 0.6253 | 4.000 |
|  | 96 | No | 0.823501 | 0.5897 | 0.7202 | -0.1305 | 0.5480 | 0.2381 | 4.000 |
| ***ORF 005L*** | 24 | No | >0.999999 | 1.000 | 1.000 | 0.000 | 0.4241 | 0.000 | 4.000 |
|  | 48 | No | 0.380125 | 1.962 | 1.413 | 0.5492 | 0.5573 | 0.9856 | 4.000 |
|  | 72 | **Yes** | 0.000227 | 10.26 | 3.927 | 6.328 | 0.5015 | 12.62 | 4.000 |
|  | 96 | No | 0.883594 | 0.8073 | 0.9177 | -0.1105 | 0.7081 | 0.1560 | 4.000 |

Statistical significance determined without correction for multiple comparisons, with alpha=0.05. Each row was analyzed individually, without assuming a consistent SD. Number of t tests: 4.

**Table S6. Test of significance for HSP gene expression of “Treated” with *ORF 005L* gene expression of “Treated” groups, and HSP gene expression of “Treated” with extracellular viral titres of “Treated” groups using the multiple t test statistical method for grouped data. (N=6)**

| **Gene** | **Time**  **point** | **Significant?** | ***P-value*** | **x̄ fold change of *HSP 90* Treated** | **x̄ fold change of *ORF005L***  **Treated** | **Difference** | **SE of Difference** | **t ratio** | **df** |
| --- | --- | --- | --- | --- | --- | --- | --- | --- | --- |
| ***HSP 90***  ***vs***  ***ORF 005L*** | 24 | No | >0.999999 | 1.000 | 1.000 | 0.000 | 0.4898 | 0.000 | 4.000 |
|  | 48 | **Yes** | 0.004624 | 4.271 | 1.413 | 2.858 | 0.4997 | 5.719 | 4.000 |
|  | 72 | **Yes** | 0.007041 | 1.481 | 3.927 | -2.446 | 0.4808 | 5.088 | 4.000 |
|  | 96 | No | 0.144106 | 1.932 | 0.9177 | 1.014 | 0.5595 | 1.813 | 4.000 |
| **HSP47**  ***vs***  ***ORF 005L*** | 24 | No | >0.999999 | 1.000 | 1.000 | 0.000 | 0.3442 | 0.000 | 4.000 |
|  | 48 | **Yes** | 0.002998 | 6.042 | 1.413 | 4.630 | 0.7193 | 6.436 | 4.000 |
|  | 72 | No | 0.179486 | 2.331 | 3.927 | -1.596 | 0.9824 | 1.625 | 4.000 |
|  | 96 | No | 0.159709 | 2.326 | 0.9177 | 1.409 | 0.8169 | 1.724 | 4.000 |
| ***HSP90***  **vs**  **Viral titre** | 24 | **Yes** | 0.000018 | 1.000 | 6.158 | -5.158 | 0.2142 | 24.08 | 4.000 |
|  | 48 | **Yes** | 0.022015 | 4.271 | 5.568 | -1.297 | 0.3566 | 3.637 | 4.000 |
|  | 72 | **Yes** | 0.000383 | 1.481 | 5.923 | -4.442 | 0.4025 | 11.04 | 4.000 |
|  | 96 | **Yes** | 0.001339 | 1.932 | 6.248 | -4.316 | 0.5410 | 7.977 | 4.000 |
| ***HSP47***  **vs**  **Viral titre** | 24 | **Yes** | 0.002284 | 1.000 | 1440260 | -1440259 | 208008 | 6.924 | 4.000 |
|  | 48 | **Yes** | 0.001296 | 6.042 | 369970 | -369964 | 45984 | 8.046 | 4.000 |
|  | 72 | **Yes** | <0.000001 | 2.331 | 837460 | -837458 | 6302 | 132.9 | 4.000 |
|  | 96 | **Yes** | 0.000792 | 2.326 | 1768749 | -1768747 | 193320 | 9.149 | 4.000 |

Statistical significance determined without correction for multiple comparisons, with alpha=0.05. Each row was analyzed individually, without assuming a consistent SD. Number of t tests: 4.
