# Supplementary material for: Investigating the mechanism of heat-shock protection in ISKNV-infected tilapia brain cell line
Source: Microbiol Spectr. 2025 Aug 12;13(9):e02510-24. doi: 10.1128/spectrum.02510-24 (PMC12403615; doi:10.1128/spectrum.02510-24)
Supplement: Figures S1 and S2 — Extrapolation of viral copy numbers and ISKNV infected TiB and BF2 cells at different time points. [file spectrum.02510-24-s0002.docx]

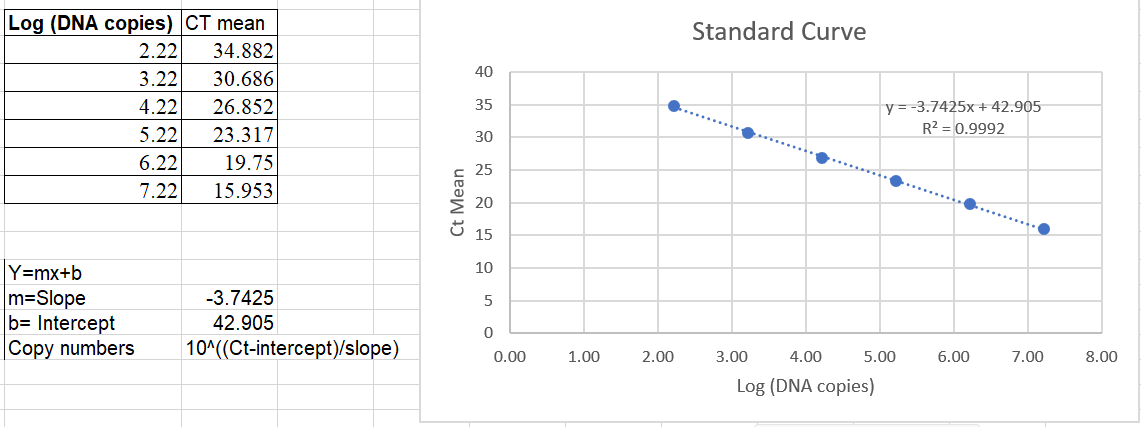


### Figure S1. Extrapolating copy numbers using the formula =>10^((observed Ct Mean-Y Intercept)/(Slope)) where intercept= 42.905 and Slope= -3.743

**
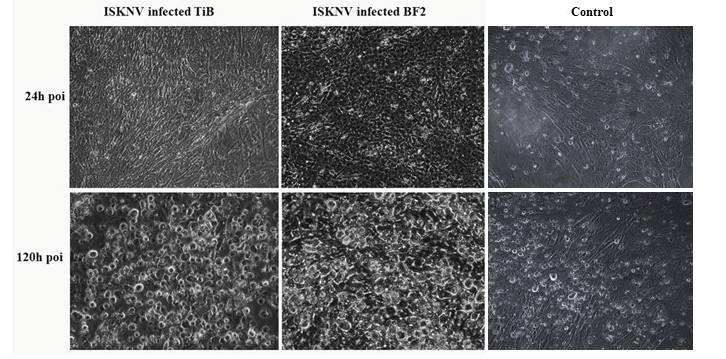
**

**Figure S2.** Images of ISKNV infected TiB and BF2 cells at 24h and 120h poi. TiB cells were completely detached by day 5 as compared to BF2 where many cells were still attached.
